# Asymmetric allelic introgression across a hybrid zone of the coal tit (*Periparus ater*) in the central Himalayas*

**DOI:** 10.1002/ece3.8369

**Published:** 2021-11-24

**Authors:** Hannes Wolfgramm, Jochen Martens, Till Töpfer, Melita Vamberger, Abhinaya Pathak, Heiko Stuckas, Martin Päckert

**Affiliations:** ^1^ Senckenberg Natural History Collections Dresden Dresden Germany; ^2^ Institute of Organismic and Molecular Evolution (iomE) Johannes Gutenberg University Mainz Germany; ^3^ Leibniz Institute for the Analysis of Biodiversity Change Zoological Research Museum Alexander Koenig Bonn Germany; ^4^ Department of National Parks and Wildlife Conservation Kathmandu Nepal; ^5^ Present address: Department of Functional Genomics Interfaculty Institute of Genetics and Functional Genomics University Medicine Greifswald Greifswald Germany

**Keywords:** birds, cline analysis, hybridization, microsatellites, mitochondrial DNA, Nepal

## Abstract

In the Himalayas, a number of secondary contact zones have been described for vicariant vertebrate taxa. However, analyses of genetic divergence and admixture are missing for most of these examples. In this study, we provide a population genetic analysis for the coal tit (*Periparus ater*) hybrid zone in Nepal. Intermediate phenotypes between the distinctive western “spot‐winged tit” (*P. a. melanolophus*) and Eastern Himalayan coal tits (*P. a. aemodius*) occur across a narrow range of <100 km in western Nepal. As a peculiarity, another distinctive cinnamon‐bellied form is known from a single population so far. Genetic admixture of western and eastern mitochondrial lineages was restricted to the narrow zone of phenotypically intermediate populations. The cline width was estimated 46 km only with a center close to the population of the cinnamon‐bellied phenotype. In contrast, allelic introgression of microsatellite loci was asymmetrical from eastern *P. a. aemodius* into far western populations of phenotypic *P. a. melanolophus* but not vice versa. Accordingly, the microsatellite cline was about 3.7 times wider than the mitochondrial one.

## Graphical abstract

In our study, we analysed patterns of divergence and admixture in the Himalayan hybrid zone of coal tits across a transect of about 1400 km from Afghanistan to East Nepal. Due to the scarcity of study material from that region only a few phylogeographic or population genetic studies exist from this area. In these cases, museum collections are a valuable source of genetic material, such as the large collections from Afghanistan and Nepal from the 1960ies to 1970ies at ZFMK Bonn used in this study.

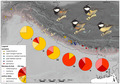

## 1 INTRODUCTION

**FIGURE 1:**
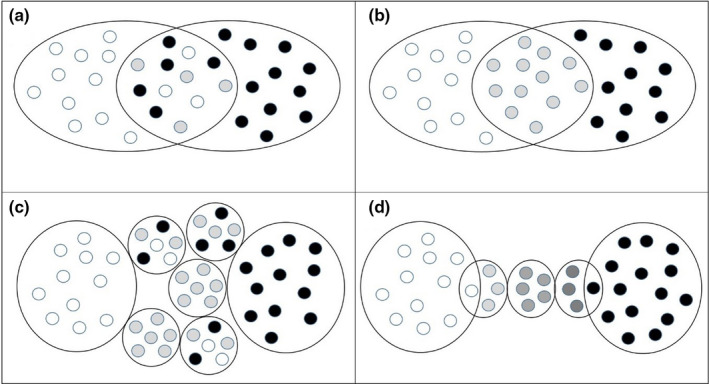
Hybrid zone models, modified and extended from Curry (2015), parental forms in black and white, hybrids in gray. (a) clinal zone, bimodal (e.g., crows, great tits); (b) clinal zone, unimodal (hybrids dominate); (c) mosaic hybrid zone (e.g., sparrows in North Africa); (d) microallopatry (e.g., coal tits in the Himalayas, this study)

Phylogeographic patterns of many Holarctic terrestrial vertebrate species are considered a legacy of Pleistocene range fragmentation and divergence of genetic lineages in glacial refuges (Hewitt, 2000, 2004, 2011; Lovette, 2005; Schmitt, 2007; Stewart et al., 2010). In southern Europe, for example, distinct mitochondrial lineages within several avian species groups can be traced back to major refugia on the Iberian, the Italian and the Balkan Peninsula as well as on some Mediterranean islands like Corsica, Sardinia, or the Balearic Islands (Brambilla et al., 2008; Brito, 2007; Nespoli et al., 2021; Pellegrino et al., 2014; Pons et al., 2016; Tritsch et al., 2018; Zuccon et al., 2020). Along with Holocene range expansion from those source areas close relatives with divergent gene pools have come into secondary contact in various zones of overlap of different extent (Aliabadian et al., 2005; Avise & Walker, 1998; Haffer, 1989). Patterns of genetic variation (e.g., divergence and gene flow among parental taxa, local admixture, etc.) can be diverse and depend, for example, on the spatial extent of range overlap, local abundances of parental taxa and hybrids as well as on strength and directionality of selective pressures acting on phenotypical or behavioral traits and on neutral and adaptive genetic variation (Curry, 2015; Jiggins & Mallet, 2000; Joseph, 2018). Generally, models for a clinal hybrid zone distinguish between (i) bimodal distributions of parental phenotypes and genotypes dominating due to strong selection against hybrids (Figure 1a) and (ii) unimodal distributions of phenotypes with hybrids dominating because of selective advantages of hybrids (Figure 1b). However, it must be stressed that distributions can strongly differ for distinct phenotypic traits, behavioral traits or different genetic markers in the same hybrid zone (Gay et al., 2007; Shipilina et al., 2017). Unimodal distributions of phenotypes are characteristic for wide areas of gene flow and phenotypic intergradation between subspecific taxa (e.g., from European birds and mammals in Hermansen et al., 2011; Pentzold et al., 2013; Smadja et al., 2003; Tritsch et al., 2018). A rare pattern is that of a mosaic hybrid zone with patchy distributions of parental taxa and hybrids in different local communities (Figure 1c), such as in the North African area of overlap between the house sparrow, *Passer domesticus*, and the Spanish sparrow, *P. hispaniolensis* (Belkacem et al., 2016; Päckert et al., 2019).

In many terrestrial vertebrates of Eurasia, such contact zones typically correspond to biogeographic barriers such as large mountain chains, for example, the Pyrenees (birds: Helbig et al., 2001; Pons et al., 2019; reptiles: Milá et al., 2013; Pöschel et al., 2018), the Alps (birds: Hermansen et al., 2011; toads: Arntzen et al., 2017; rodents: Giménez et al., 2017; Sutter et al., 2013), the Carpathians (newts: Babik et al., 2003; Gherghel et al., 2012; toads: Fijarczyk et al., 2011; Hofman et al., 2007), and the Urals (birds: Shipilina et al., 2017). Contact zones between northern and southern lineages can occur in more than one mountain range, such as the capercaillie (*Tetrao urogallus*) in the Pyrenees, the Dinaric mountains and the Carpathians (Bajc et al., 2011; Segelbacher & Piertney, 2007). As the largest Eurasian mountain system with the highest peaks on Earth the Himalayas are a prominent global biodiversity hotspot (Marchese, 2015; Martens, 2015; Myers, 2003; Myers et al., 2000). Its local and regional faunal and floral assemblages have long and complex evolutionary and biogeographic histories including both in‐situ speciation and immigration from adjacent regions (Favre et al., 2015; Martens, 2015; Mosbrugger et al., 2018; Päckert et al., 2020). While past diversification has been subject to a great number of studies, extant patterns of distribution and gene flow in secondary range overlap for the Himalayan fauna have been less intensely studied to date.

**FIGURE 2:**
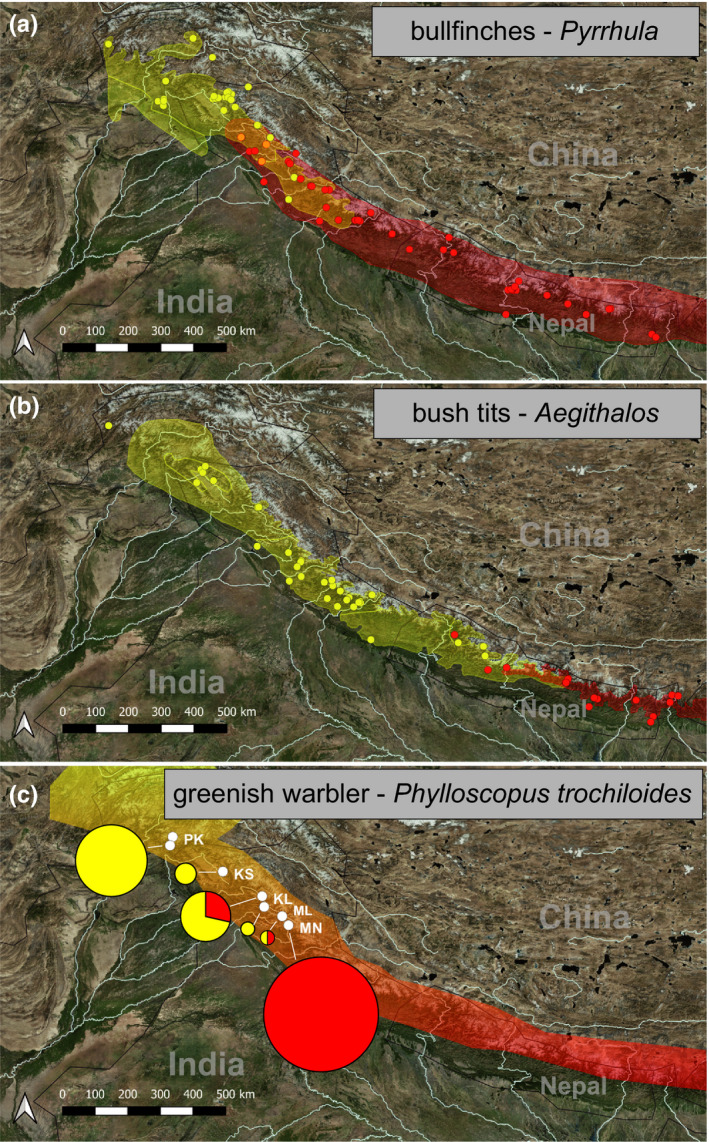
Areas of secondary overlap in the Western Himalayas for three passerine taxon pairs, each represented by two distinct genetic mtDNA lineages (western = yellow; eastern = red). (a) orange bullfinch, *Pyrrhula aurantiaca*, and red‐headed bullfinch, *P. erythrocephala* (data from eBird, 2020; Töpfer et al., 2011; Wunderlich, 1992a, 1992b), local sympatry: orange circles); (b) white‐throated bushtit, *Aegithalos niveogularis*, and black‐browed tit, *Ae. iouschistos* (data from eBird, 2020; Päckert et al., 2010; Wunderlich, 1989, 1991); (c) greenish warbler, *P. trochiloides*, pie charts show local frequencies of haplotypes from the eastern (*P. t. trochiloides*: red) and western (*P. t. ludlowi*: yellow) mtDNA lineage; data modified from Irwin et al. (2001) and Irwin et al. (2005); distribution shape files from BirdLife International (2020); Shape file for *P. trochiloides* modified according to Irwin et al. (2001) and Irwin et al. (2005); maps produced with QGIS v. 3.10

**FIGURE 3:**
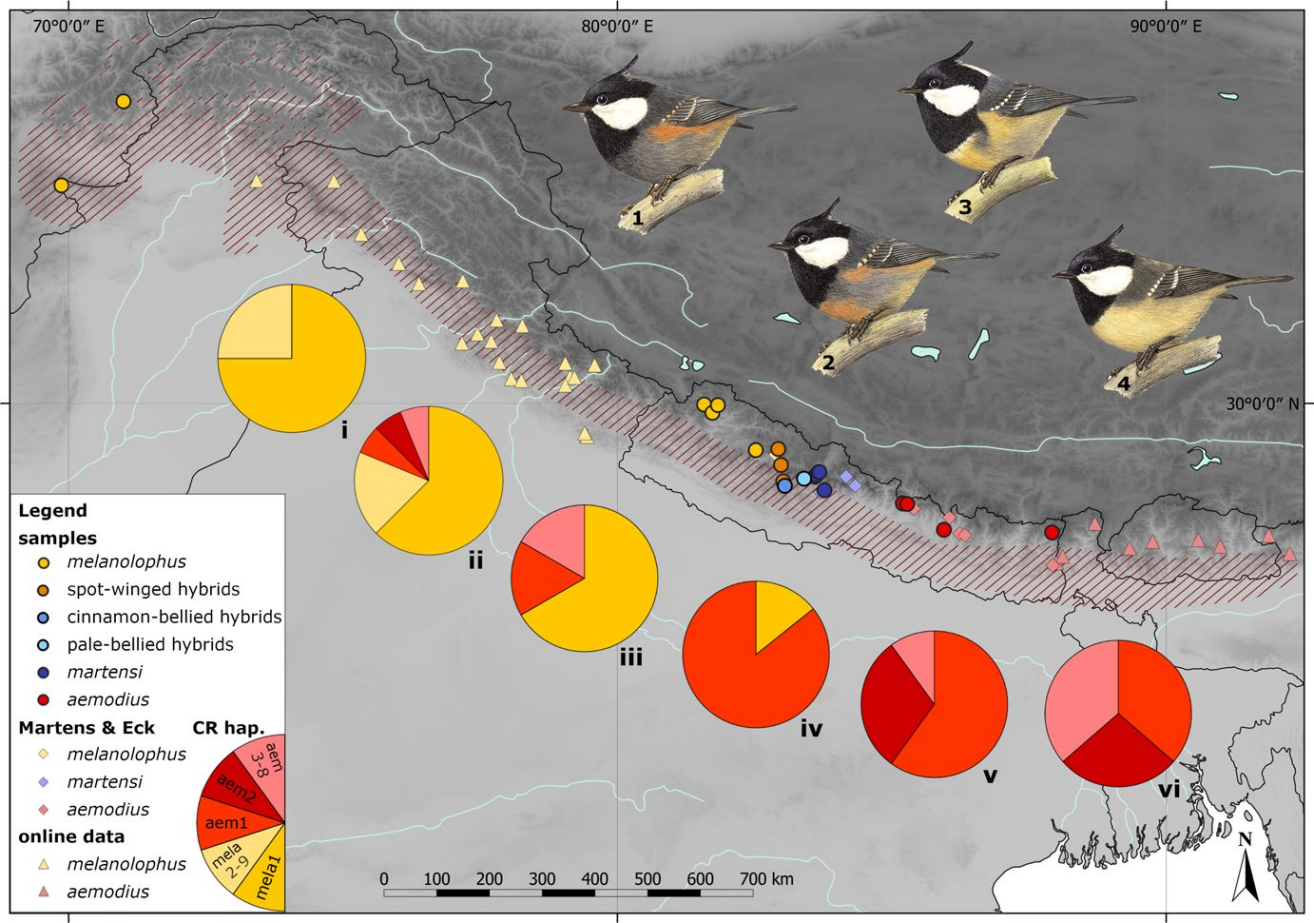
The Himalayan contact zone of western and eastern subspecific taxa of the coal tit. Parental taxa: *Periparus ater melanolophus* (phenotype 1, population i) and *P. a. aemodius* (phenotype 4, population vi); pie charts show local frequencies of CR haplotypes from the western (yellow; *n* = 9) and eastern (red; *n* = 8) lineages (compare Figure 4); putative hybrid populations with intermediate phenotypes exist in a narrow area of overlap in Central Nepal (from west to east): spot‐winged‐type hybrids (population ii), cinnamon‐bellied hybrids (phenotype 2, population iii, occurrence at Dhorpatan in local sympatry with the spot‐winged‐type), pale‐bellied hybrids (population iv, at Myagdi Khola) and *P. a. martensi* (phenotype 3, population v); symbolization: brightly‐colored dots = own samples, pale dots = records by Martens and Eck (1995), triangles = data from online databases (sound recordings from xeno‐canto, 2017; photographs revisited at Oriental Bird Club, 2017), red‐shaded area = distribution according to BirdLife International (2021); drawings by K. Rehbinder

Along the Himalayan mountain chain eastwest vicariance is typically found for many avian taxon pairs regardless of their taxonomic rank at the species or subspecies level (Martens, 2015; Martens et al., 2011; Päckert et al., 2011, 2015). These Himalayan vicariants typically (i) diverged during the early or mid‐Pleistocene and (ii) meet in narrow zones of range overlap in secondary contact (e.g., in Figure 2). Despite a considerable knowledge gain on the genetic diversification of Himalayan birds there is still a great deficiency of field data and therefore the extent of putative zones of overlap remain poorly described to date for many Himalayan taxon pairs. Several areas of secondary overlap and gene flow among vicariant vertebrate taxa were described from the western Himalayas (Figure 2; Maheshwari et al., 2013). For Nepal, Martens and Eck (1995) defined four subspecies transition areas, where western and eastern vicariants of the same species co‐occur in secondary contact. Across the Dhaulagiri transition zone (Figure S1, Martens & Eck, 1995) extends a narrow belt of putative hybrid populations between two subspecies of the the coal tit: the western dark‐bellied and red‐flanked form *P. a. melanolophus* (Figure 3, phenotype 1) and the eastern pale‐bellied form *P. a. aemodius* (Figure 3, phenotype 4). These intermediate phenotypes were already described in the 1970s based on morphology and territorial songs (Diesselhorst & Martens, 1972; Martens, 1975). At its easternmost range margins on the southwestern slopes of the Dhaulagiri massif (Parbat and Dolpa Districts), local aberrant plumage color variants of *P. a. melanolophus* (so called “spot‐winged‐type hybrids”; Harrap & Quinn, 1996) were suggested to have originated from hybridization with eastern pale‐bellied coal tit populations (Diesselhorst & Martens, 1972). As a peculiarity of the hybrid zone, another very distinctive phenotype occurs only locally: from Dhorpatan Valley (Baglung District) a cinnamon‐bellied form was documented that occurs in local syntopy with the latter “spot‐winged‐type hybrids” (Figure 3, phenotype 2; Diesselhorst & Martens, 1972; Eck & Martens, 2006; Martens, 1975; Martens & Eck, 1995). A suspected hybrid origin of these cinnamon‐bellied birds received further support from cross‐fostering experiments by Löhrl (1994) whose F1 and F2 hybrids *P. a. melanolophus* × *P. a. ater* showed that aberrant phenotype, too. In western Nepal, pale‐bellied populations from the upper Kali Gandaki Valley (Figure 3, phenotype 3) were classified as “coal‐type hybrids” by Harrap and Quinn (1996: Figure 63.2) but later described as a distinct subspecies *P. a. martensi* by Eck (1998) based on morphological differences from *P. a. aemodius*. From the upper Myagdi Khola Martens and Eck (1995) described local intermediate phenotypes between *P. a. martensi* and *P. a. melanolophus* and first genetic analyses by Martens et al. (2006) confirmed that specimes from this population disposed of either of two separate parental mitochondrial lineages. However, their sampling included only four putative hybrid individuals.

Each of those diverse local coal tit phenotypes is restricted to narrow and isolated breeding areas in separate steep mountain valleys of central Himalayan river catchments (Figure 3; Diesselhorst & Martens, 1972; Martens, 1975; Martens & Eck, 1995). Thus, according to phenotypical variation the spatial pattern in the Himalayan coal tit hybrid zone is one of microallopatry (Figure 1d; according to a geographical concept instead of an ecological concept; see review by Fitzpatrick et al., 2008) comparable to that of other montane taxa like Himalayan ground beetles (genus *Ethira*; Schmidt et al., 2012) or *Buthus* scorpions of the Atlas Mountains in North Africa (Habel et al., 2012).

With this study, we describe patterns of genetic divergence and admixture of Himalayan coal tit populations across a wide transect from the Hindukush in the West to eastern Nepal in the East. We expect (i) strong genetic admixture of phenotypically intermediate populations in western Nepal (Figure 3, blue marked populations) and (ii) genetic distinctiveness of putative parental populations at both ends of the phenotypic cline, that is, *P. a. melanolophus* in the Western Himalayas and its extensions to the Hindukush and *P. a. aemodius* in eastern Nepal. The study material is largely based on historical samplings from natural history museums collected during the 1960s and the 1970s emphasizing the importance of collections as biological archives (Kuhn et al., 2013; Mecke et al., 2016; Meineke et al., 2018; Rocha et al., 2014; Winston, 2007).

## 2 MATERIALS AND METHODS

### 2.1 Sampling and DNA extraction

We analyzed 70 coal tit samples from 20 localities across a transect from Afghanistan in the West (*P. a. melanolophus*) to eastern Nepal in the East (*P. a. aemodius*; Figure 3; Table 1). Material from Nepal was mainly collected by J.M. during five expeditions in 1969/1970 and 2004 and most specimens analyzed are housed at Zoological Research Museum Koenig Bonn (ZFMK) and Naturkundemuseum Erfurt (see Table S1). Our sampling included the following phenotypically distinct populations (morphological diagnosis in Martens & Eck, 1995): *P. a. melanolophus* (*n* = 20), *P. a. martensi* (*n* = 10), *P. a. aemodius* (*n* = 11), cinnamon‐bellied hybrids (*n* = 6), pale‐bellied hybrids (*n* = 7) spot‐winged type hybrids (*n* = 16). We used DNA extracts from previous studies (e.g., Pentzold et al., 2013) and newly extracted DNA from additional frozen blood and muscle tissue samples using innuPREP DNA Mini Kit™ (Analytik Jena, Jena, D). Further additional toe pad samples from historical specimens collected during the 1970s were processed in a separate clean room facility to avoid cross contamination with DNA from fresh samples (for specification of lab protocols in clean room facilities see Tritsch et al., 2018). DNA from toe pads was extracted using sbeadex™ forensic Kit (LGC, Teddington, UK) with an extraction volume of 75 µl elution buffer (see Tritsch et al., 2018).

**TABLE 1:** Coal tit samples analyzed in this study

Taxon	*n*	Sample IDs	Country	Province	Locality	Latitude	Longitude
*Periparus ater melanolophus*	10	MAR8322 – MAR8329, MAR8332 – MAR8333	Afghanistan		Peiwar	33.9667	69.8667
*Periparus ater melanolophus*	3	MAR8330 – MAR 8331 MTDC52891	Afghanistan		Sinsoi/Nuristan	36.1931	71.0000
*Periparus ater melanolophus*	1	MTDC52890	Afghanistan				
*Periparus ater melanolophus*	1	MAR2913	Nepal	Humla District	Chala, valley below	29.9833	81.5833
*Periparus ater melanolophus*	1	MAR2921	Nepal	Humla District	Simikot, W of	29.9667	81.8167
*Periparus ater melanolophus*	1	MAR3324	Nepal	Humla District	Chucho Khola	29.9667	81.8333
*Periparus ater melanolophus*	1	MAR2918	Nepal	Humla District	Saipal	29.8333	81.7333
*Periparus ater melanolophus*	1	MAR2959	Nepal	Humla District			
*Periparus ater melanolophus*	1	MAR6615	Nepal	Jumla District	Jagdula Lekh valley	29.1500	82.5167
*P. a. melanolophus* × *P. a. martensi* (spot‐winged‐type)	1	MAR8316	Nepal	Dolpa District	Ringmo at Phoksumdo lake	29.1667	82.9333
*P. a. melanolophus* × *P. a. martensi* (spot‐winged‐type)	2	MAR8317 – MAR8318	Nepal	Dolpa District	Gompa, Tarakot	28.8833	82.9833
*P. a. melanolophus* × *P. a. martensi* (spot‐winged‐type)	3	MAR8308 MAR8314 – MAR8315	Nepal	Baglung District	Thankur	28.5833	83.0167
*P. a. melanolophus* × *P. a. martensi* (spot‐winged‐type)	10	MAR8306 – MAR8307, MAR8309 – MAR8313 MAR8319 – MAR8321	Nepal	Baglung District	Dhorpatan valley	28.5000	83.0500
*P. a. melanolophus* × *P. a. martensi* (cinnamon‐bellied type)	6	MAR8301 – MAR8305 MTDC58572	Nepal	Baglung District	Dhorpatan valley	28.5000	83.0500
*P. a. melanolophus* × *P. a. martensi* (pale‐bellied type)	7	MAR90155 – MAR90157, MAR90163 – MAR90166	Nepal	Myagdi District	upper Myagdi Khola	28.3833	83.5500
*Periparus ater martensi*	1	MAR8335	Nepal	Mustang District	Nabrikot Khola	28.6667	83.6000
*Periparus ater martensi*	3	MAR8336, MAR8338, MAR8340	Nepal	Mustang District	Kali Gandaki valley, Thaksang, above Tukche	28.7000	83.6333
*Periparus ater martensi*	2	MAR90132, MAR90137	Nepal	Mustang District	Purano Marpha	28.7500	83.6667
*Periparus ater martensi*	3	MAR8334, MAR8337, MAR8339	Nepal	Mustang District	Thakkhola	28.7500	83.6800
*Periparus ater martensi*	1	MAR90101	Nepal	Parbat District	Marsyandi, between Chitre and Deorali	28.4167	83.7667
*Periparus ater aemodius*	3	MAR4155 – MAR4156, MAR4222	Nepal	Rasuwa District	Somdang, W Syabrubesi, 4. & 8. camp	28.1833	85.2000
*Periparus ater aemodius*	2	MAR4195 – MAR4196	Nepal	Rasuwa District	Somdang, W Syabrubesi, 6. camp	28.1667	85.1833
*Periparus ater aemodius*	5	MAR90018 – MAR90019, MAR90028, MAR90046, MAR90053	Nepal	Sindhupalchok District	Dadar Danda, Kalinchok	27.7000	85.9500
*Periparus ater aemodius*	1	MAR3681	Nepal	Taplejung District	Camp below Ghunsa	27.6500	87.9167

Note

Information on the sampling including phenotypic classification, sample size per locality, sample IDs, and detailed locality coordinates.

### 2.2 Mitochondrial DNA

We amplified a fragment covering the first domain and parts of the second domain of the mitochondrial control region (CR) from frozen tissue samples using primers L16700 (5′‐ATCATAAATTCTCGCCGGGACTCT‐3′) and H636 (5′‐GAGATGAGGAGTATTCAACCGAC‐3′; both from Kvist et al., 2003) following lab protocols of Pentzold et al. (2013). For amplification of two shorter fragments with degraded DNA from toe pad extracts we used OligoAnalyzer v. 1.0.2 for design of two internal primers PeripCR_183f (3′‐ACGCCCAAGAGATAATGTTCG‐5′) combined with H636 and PeripCR_451r (3′‐AGGTCCTCTGGCTTGGG‐5′) combined with L16700. In order to determine the optimum annealing temperature for each primer pair we performed gradient PCRs. According to this a‐priori evaluation, the PCR profile for amplification of the short fragments was (i) denaturation at 94°C for 5 min followed by 30 cycles of (ii) denaturation at 94°C for 45 s, (iii) annealing at 53°C for 45 s, and (iv) elongation at 72°C for 1 min with final elongation phase at 72°C for 8 min. PCRs for DNA extracts from toe pad samples were prepared in the clean‐room facility (for protocols see Tritsch et al., 2018). PCR products were purified in an enzymatic reaction using ExoSAPit and sequenced on an ABI 3130xl capillary sequencer (Applied Biosystems™).

We aligned CR sequences using ClustalW as implemented in MEGA 5.1 (Tamura et al., 2011) and checked chromatograms for potential sequencing errors such as double peaks with Chromas lite (Technelysium Pty Ltd). For comparison we added CR sequences from Chinese populations (*P. a. eckodedicatus*) and Far East Russian populations (*P. a. ater*) from Pentzold et al. (2013). For Genbank accession numbers of newly generated sequences and those inferred from previous studies, see Tables S1 and S2. Because amplification of the first CR fragment (primers L16700 + PeripCR_451r) performed poorly for a considerable number of toe pad samples, we used only the second 437 bp long fragment for analysis (inferred from amplification with primers PeripCR_183f and H636). To ensure that all sequences had the same length, we had to cut down the alignment to 324 base pairs. We used PopArt v1.7 (Leigh & Bryant, 2015) for construction of a minimum‐spanning haplotype network (Bandelt et al., 1999) of CR sequences.

Furthermore, we reconstructed a time‐calibrated phylogeny using BEAST v.1.8.1 (Drummond et al., 2012). For hierarchical outgroup rooting, we used one sequence of the yellow‐bellied tit, *Pardaliparus venustulus* (inferred from a mitochondrial genome: NC_026701), and another sequence of the blue tit, *Cyanistes caeruleus* (JF828052) as a more distantly related relative. The best‐fit model estimated with MrModeltest v.2 (Nylander, 2004) for our data set was the K80 + G model with equal base frequencies (according to the Akaike Information Criterion, AICc). According to these model estimates, we applied normal priors to kappa (mean: 2.2465, *SD*: 0.2) and to the gamma‐shape parameter (mean: 0.3027, *SD*: 0.03). For inference of divergence times estimates, we applied a molecular clock calibration using mean substitution rates for different domains of the CR estimated by Lerner et al. (2011) for Hawaiian honeycreepers (Drepanidinae). Their rate estimates for the CR ranged between 0.011 and 0.029 substitutions per site per lineage per Million years, so we applied a mean normal prior of 0.2 and a standard deviation of 0.2 to adjust the 95% CI of the rate prior to that range. We ran BEAST for 50 million generations (with trees sampled every 5000 generation) under the uncorrelated lognormal clock model with the “auto‐optimize” option activated and a Yule prior applied to the trees. We checked for convergence of MCMC chains comparing ESS values for all model parameters using Tracer v. 1.4 (all ESS values > 4.000; Rambaut & Drummond, 2007). We used TreeAnnotator for reconstruction of a consensus tree with a burnin of 30% applied and we used FigTree v. 1.4.2 (Rambaut, 2009) for editing of the Bayesian tree.

For each population we calculated diversity estimates like number of haplotypes (h), haplotype diversity (Hd), nucleotide diversity (π), and Tajima's D with DNASP v. 5.10.01 (Librado & Rozas, 2009). Divergence between populations was estimated by calculating pairwise F_ST_ values using ARLEQUIN 3.5.1.3 (Excoffier et al., 2005) with 20,000 permutations to test for significance. All *p*‐values from multiple comparisons were adjusted using the Bonferroni correction to reestimate the significance level (Rice, 1989).

### 2.3 Microsatellite genotyping

We performed pilot analyses using a set of 13 microsatellite loci developed for European coal tit populations by Tritsch et al. (2018). We designed a new multiplex microsatellite protocol based on fragment length variation evaluated in the previous study (Tritsch et al., 2018). To maximize spacing between markers with overlapping fluorescence spectra (Guichoux et al., 2011), we divided the primer pairs into two separate multiplex sets (Table S3). For multiplex PCR, we used the Type‐it^®^ Microsatellite PCR Kit (Qiagen) following the manufacturer's instructions. For each of the two multiplex approaches a primer premix was prepared containing 10 µl primer solution (10 ng/µl) for each primer filled up to a total volume of 500 µl. For each sample, a total multiplex reaction volume of 13.5 µl contained 6.25 µl Master‐Mix (Type‐it Kit), 1.25 µl primer mix, 4 µl ddH_2_O, and 2 µl DNA. The thermo‐cycling protocol for both multiplex sets was (i) denaturation at 95°C for 5 min followed by 30 cycles for fresh DNA extracts (35 cycles for toe pad DNA extracts) with (ii) denaturation at 95°C for 30 s, (iii) annealing at 56°C for 1 min 30 s, and (iv) elongation at 72°C for 45 s and a final elongation phase at 60°C for 30 min. Fragment length analysis was performed on a 16‐column ABI 3130xl capillary sequencer (Applied Biosystems™) for total volumes of 10 µl containing 1 µl diluted muliplex PCR products (1:10 for toe pad samples and 1:25 for frozen tissue/blood samples), 8.5 µl Hi‐Di™ Formamid (Applied Biosystems™), 0.25 μl GeneScan™‐600 LIZ^®^ dye size standard, and 0.25 μl ddH_2_O.

In the few cases, when the multiplex PCR failed for a single locus (in most cases the longer fragments failed to amplify) we repeated the microsatellite genotyping for that locus in a separate PCR to account for possible allelic dropout that can typically occur with degraded DNA from historical samples (Sefc et al., 2003). Results were significantly improved for most samples and loci except three loci Pma69, PmaC25, and PmaTGAn33 that produced missing or ambiguous signal for a larger number of samples. Due to this data deficiency and because these loci were originally designed for great tit (*Parus major*) populations (Kawano, 2003; Saladin et al., 2003) these three loci were discarded from further analyses.

Alleles were scored manually using Peak Scanner™ Software Version 1.0 (Applied Biosystems™). We converted raw allele size data from Excel sheets to generate input files for various population genetic software packages using CONVERT v. 1.31 (Glaubitz, 2004) and PGDSPIDER v. 2.1.1.0 (Lischer & Excoffier, 2012). A data package including microsatellite allele lengths and the CR alignment was deposited at Dryad Digital Repository and is available under https://doi.org/10.5061/dryad.0gb5mkm28.

We used MICROCHECKER v. 2.2.3 (van Oosterhout et al., 2004) to test for the presence of null alleles (Falush et al., 2007) and possible allele scoring errors due to the presence of stutter bands. We tested for locus specific deviations from Hardy Weinberg expectations (HWE) and for linkage bewteen loci with ARLEQUIN v. 3.5.1.3. Deviations from HWE and presence of null alleles were predominately found at loci Parate06 and Parate08 in three and five populations, respectively (Table S4). For these two loci similar deviations from HWE were found in a previous study on the European zone of gene flow and introgression of the coal tit (Tritsch et al., 2018) suggesting that these loci should be treated precautiously. Therefore, we performed most downstream analysis twice, for both the entire set of 10 loci and for a reduced set of 8 loci under exclusion of Parate06 and Parate08. Quantitative diversity and divergence estimates were calculated for eight loci only (Parate06 and Parate08 excluded). Further deviations from HWE and null alleles appeared only in the populations of the spot‐winged‐type hybrids and *P. a. melanolophus* for individual loci. In these western populations as well as in the eastern population of *P. a. aemodius*, two and three pairs of loci, respectively, were also found in pairwise linkage disequilibrium (Table S4). It should be noted that these deviations from the HWE might be caused by the genetic structure of the populations as well as by the small sample size.

Due to low sample sizes for local populations, we pooled our samplings according to the distinct phenotypes in the zone of overlap and compared six metapopulations from west to east (Figure 3, populations i–vi): (1) *P. a. melanolophus* from Afghanistan and mid‐western Nepal, (2) spot‐winged‐type hybrids from Dolpa and Baglung Districts (locally sympatric with cinnamon‐bellied hybrids), (3) cinnamon‐bellied hybrids from Dhorpatan, (4) pale‐bellied hybrids from Myagdi District, (5) *P. a. martensi* from Mustang and Parbat Districts, (6) *P. a. aemodius* from Rasuwa and Sindhupalchok Districts. We calculated locus specific observed and expected heterozygosities (H_O_, H_E_), mean allele numbers per locus, mean allelic richness (AR) and inbreeding coefficients (F_IS_) for each sample population with the software FSTAT v. 2.9.3.2 (Goudet, 1995); 1200 permutations were performed in a randomization test for significance of these values. Divergence between populations was estimated by calculating pairwise F_ST_ values using ARLEQUIN 3.5.1.3 with 20,000 permutations to test for significance. All *p*‐values from multiple comparisons were adjusted using the Bonferroni correction to re‐estimate the significance level (Rice, 1989).

### 2.4 Inference of population structure

Bayesian inference of population structure was performed using the software package STRUCTURE v. 2.3.3. (Falush et al., 2003; Pritchard et al., 2000). STRUCTURE runs were performed for both the entire set of 10 loci and the reduced set of 8 loci under (i) the a priori assumption of genetic admixture and correlated allele frequencies and (ii) a LOCPRIOR model allows for classification of the individuals into groups, which are given to the algorithm as an a priori parameter (Hubisz et al., 2009). All STRUCTURE runs were conducted for 1–10 putative genetic clusters (K) with 10 replicates for each value of K. We used a MCMC chain length of 10^6^ repetitions with a burn‐in period of 25,000 throughout all model runs. For further processing of the output, we used STRUCTURE HARVESTER (Earl & vonHoldt, 2012), results were visualized using DISTRUCT (Rosenberg, 2004). In order to select the most likely number of genetic clusters (K), we followed the approach by Evanno et al. (2005). As an estimate for the extent of genetic admixture in different populations we adhered to the approach by Randi (2008) and used a threshold of q > 0.8 for individual assignment probability to one cluster. Individuals with inferred q scores between 0.2 ≤ q ≤ 0.8 as well as individuals showing mitonuclear discordance (cluster assignment according to mitochondrial CR haplotype and q score is contradictory) are considered hybrids.

In addition to STRUCTURE analyses, we examined our microsatellite data with Principal Component Analysis (PCA) using the R package *adegenet* (Jombart, 2008; Jombart & Ahmed, 2011) executed in R version 3.2.3 (R Core Team, 2015) for three groups: (1) western lineage: *P. a. melanolophus* from Afghanistan and mid‐western Nepal, (2) eastern lineage: *P. a. aemodius* and *P. a. martensi*, and (3) admixed: all phenotypic hybrids.

### 2.5 Geographic cline analysis

To explore the extent of the genetic cline in the Himalayas, maximum‐likelihood cline models were generated in the R package HZAR (Derryberry et al., 2014). We applied the example script “Data S1” with modifications according to Stuckas et al. (2017) executed in R version 3.4.1 (R Core Team, 2020) in the RStudio environment (RStudio Team, 2020). In HZAR, 15 model variants can be fitted, varying in the combination of three possible scaling parameters (assignment probability at the transect ends *p*_min_ and *p*_max_ fixed at 0 and 1, set to estimated values or fit to observed values) and five possible tail fittings (none fitted, left only, right only, mirror tails, and both tails estimated separately). Previous applications of HZAR for cline analyses focused, for example, on hybrid zones of newts (Tominaga et al., 2018), toads (Arntzen et al., 2017; van Riemsdijk et al., 2019) and butterflies (Capblancq et al., 2020). To collapse sample localities of our study in the one‐dimensional axis, we defined the cinnamon‐bellied population at Dhorpatan as the tentative center of the cline and estimated great circle geographical distances of each population from Dhorpatan. For localities west of Dhorpatan distances were expressed as negative values, for those east of Dhorpatan as positive values (Table S1). These values were transferred to positive values on a theoretical transect of 1887 km length starting at Peiwar, Afghanistan in the West and ending in Nepal at Ghunsa, Taplejung District, in the East. Clines were estimated for CR haplotype frequencies (pooled populations see Table S1) and the q score for each individual as inferred from our STRUCTURE analysis with 10 microsatellite loci for K = 2. The best‐fit model each was selected according to corrected AICc (Akaike, 2011) scores including a comparison against a null model. We extracted maximum‐likelihood width and center of the cline as well as the two log‐likelihood confidence intervals for both. Parameters were considered statistically significant to each other if the confidence intervals did not overlap.

## 3 RESULTS

**FIGURE 4:**
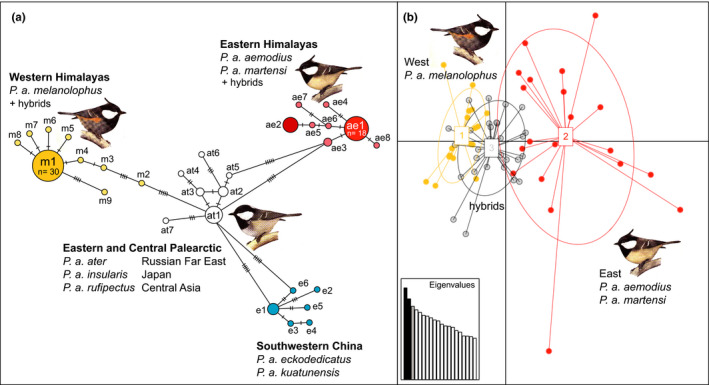
Genetic differentiation of coal tits across and beyond the Himalayan contact zone. (a) minimum‐spanning haplotype network of mtDNA CR sequences (324 bp), including mitochondrial lineages from the Himalayas (western = yellow; eastern = red) and adjacent lineages from China (blue) and from the Russian Far East (white; including sequence data from Pentzold et al., 2013); (b) PCA of microsatellite data (all ten loci; *x*‐axis = PC1, *y*‐axis = PC2; boxes = centroids for the three groups); first three principal components each explain 4.58%, 4.03% and 3.66% of variation (Eigenvalues shown in square lower left); drawings by K. Rehbinder

**FIGURE 5:**
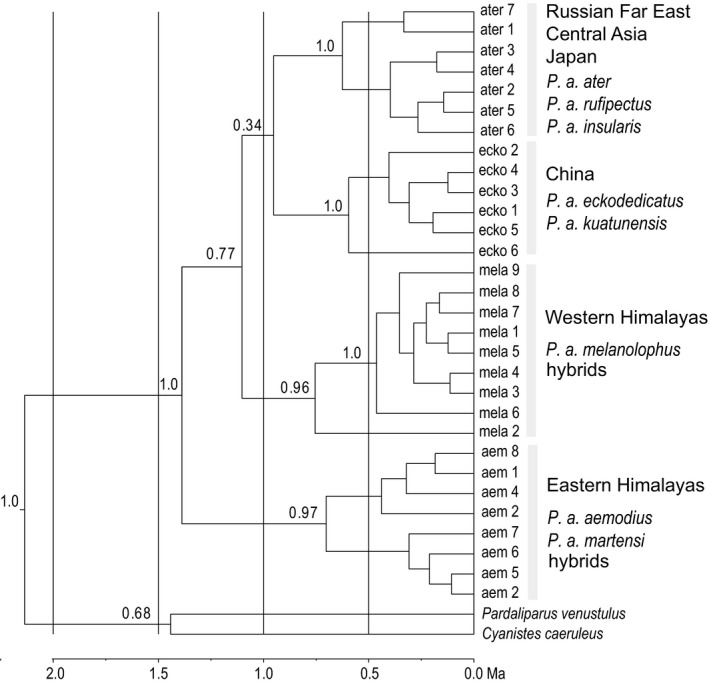
Time‐calibrated phylogeny for 30 control‐region haplotypes (324 bp) of Asian coal tit (*Periparus ater*) populations and two outgroups (*Pardaliparus venustulus*; *Cyanistes caeruleus*). Bayesian tree inferred from MCMC run with BEAST for 50 million generations, trees sampled every 5000 generation, uncorrelated lognormal clock model, Yule tree prior, burnin 30%; node support from posterior probabilities shown above nodes (except internal topology of each of the four Asian clades; most values < 0.9)

A total of 30 CR haplotypes was found in the Asian coal tit data set. The 324‐bp long alignment contained 37 variable sites of which 21 were parsimony‐informative. The minimum‐spanning network was divided into four haplotype clusters (Figure 4a) corresponding to four well supported clades in the time‐calibrated Bayesian tree (Figure 5). Three outer haplotype clusters from the Eastern and Western Himalayas and from China were separated from the central haplotype cluser of the network (Far East Russia and Central Asia) at equal distances (minimum of five substitutions; Figure 4a). The central haplotype of the starlike Western Himalayan cluster (mela1) was shared by 30 individuals (Figure 4a) and was the dominating haplotype in *P. a. melanolophus* populations from Afghanistan and from Western Nepal as well as in the cinnamon‐bellied hybrid population from Dhorpatan (Figure 3, populations i, ii, and iii). The most common haplotype of the Eastern Himalayan cluster (aem1) was shared by 18 individuals (Figure 4a) and was the dominant haplotype in populations of *P. a. martensi* and of pale‐bellied hybrids (both Myagdi District, Nepal; Figure 3, populations iv and v). According to our time calibration, the earliest split occurred between the Eastern Himalayan mitochondrial lineage and the remaining three Asian lineages during the mid‐Pleistocene at about 1.5 Ma (95% highest posterior density interval [HPDI] = [0.6–2.7 Ma]; Figure 5).

Local mitochondrial gene pools in Afghanistan and mid‐western Nepal (Humla District) comprised only haplotypes of the western *P. a. melanolophus* lineage (Figure 3, population i) and local gene pools in central and eastern Nepal (Mustang District, Parbat District, Rasuwa District, Sindhupalchok District and Taplejung District) comprised only haplotypes of the eastern *P. a. aemodius* lineage (Figure 3, populations v [*P. a. martensi*] and vi [*P. a. aemodius*]). Local admixture of the two haplotype lineages was found across all phenotypic hybrid populations from western Nepal Myagdi District (Figure 3, populations ii, iii, iv). Accordingly, nucleotide diversity was at least two times higher in admixed populations from western Nepal as compared to populations at the western and eastern ends of the Himalayan transect (Table 2). Haplotype diversity was highest in *P. a. aemodius* and rather low in allopatric *P. a. melanolophus* (lowest Hd for pale‐bellied hybrids; Table 2). Tajima's D was significantly negative for two populations only: Allopatric *P. a. melanolophus* (Afghanistan and mid‐western Nepal) and pale‐bellied hybrids from Myagdi District.

**TABLE 2:** Genetic diversity indices of Himalayan coal tit populations (*n*, number of individuals)

Pop	mtDNA (CR)						Microsatellites (8 loci)					
	*n*	h	Hd	π	Tajima‘s D	*p* (D)	*n*	AR	H_O_	H_E_	*F*_IS_	*p* (*F*_IS_)
*Periparus ater melanolophus*	20	6	0.447	0.00376	**−2.0976**	**<.05**	19	4.463	0.586	0.673	**0.134**	.**0010**
Spot‐winged‐type hybrids	16	6	0.542	0.01508	−0.23269	>.10	16	4.986	0.643	0.747	**0.144**	.**0031**
Cinnamon‐bellied hybrids	6	3	0.600	0.02484	0.90194	>.10	6	5.500	0.771	0.775	0.005	.5177
Pale‐bellied hybrids	7	2	0.286	0.01195	**−1.62257**	**<.05**	7	5.955	0.857	0.782	−0.105	.0677
*P. a. martensi*	10	3	0.600	0.00606	0.50521	>.10	10	5.667	0.685	0.790	**0.139**	.**0052**
*P. a. aemodius*	11	6	0.836	0.00730	0.49420	>.10	9	6.022	0.875	0.843	−0.040	.2698

Note

Significant values shown in bold.

mtDNA: h, number of haplotypes; Hd, haplotype diversity; π, nucleotide diversity and Tajima's D (including *p*‐value; *p*(D)); Microsatellites (8 loci, Parate06 and Parate08 excluded): AR, mean allelic richness; H_O_, mean observed heterozygosity; H_E_, mean expected heterozygosity; *F*_IS_, inbreeding coefficient (including *p*‐value; level of significance after Bonferroni correction *p* < .05/6 = .008).

Microsatellite data showed a less clear distinction of the two genetic clusters than mtDNA and suggested a broader area of admixture between western *P. a. melanolophus* and eastern *P. a. aemodius*. PCA for 10 loci distinguished two separate clusters of western and eastern parental lineages with hybrid populations from western Nepal showing a greater overlap with the western *P. a. melanolophus* cluster (Figure 4b). As to be expected F_ST_ values from mtDNA and microsatellite data were highest and significant for pairwise comparisons among westernmost and easternmost populations of *P. a. melanolophus* and *P. a. aemodius*, as well as among spot‐winged‐type hybrids and *P. a. aemodius* (Table 3). For the mtDNA dataset F_ST_ values for pairwise comparisons of *P. a. melanolophus* and *P. a. martensi*, *P. a. melanolophus* and pale‐bellied hybrids, spot‐winged‐type hybrids and *P. a. martensi*, spot‐winged‐type hybrids and pale‐bellied hybrids as well as cinnamon‐bellied hybrids and *P. a. aemodius* were significant, too (Table 3). The western *P. a. melanolophus* shows as well as the populations of *P. a. martensi* and the spot‐winged‐type hybrids significant *F*_IS_ values (Table 2).

**TABLE 3:** Pairwise *F*_ST_ values inferred from the mitochondrial DNA (CR; data set below diagonal) and inferred from the microsatellite data set (8 loci, Parate06, and Parate08 excluded; above diagonal)

	*Periparus ater melanolophus*	Spot‐winged‐type	Cinnamon‐bellied	Pale‐bellied	*Periparus ater martensi*	*Periparus ater aemodius*
*P. a. melanolophus*		0.02470	0.01662	0.04732	0.04508	**0.10819**
Spot‐winged‐type hybrids	0.07319		0.02018	0.00154	0.00967	**0.06155**
Cinnamon‐bellied hybrids	0.30171	−0.04505		0.01221	0.00998	0.04060
Pale‐bellied hybrids	**0.83856**	**0.55655**	0.33511		−0.01722	0.01946
*P. a. martensi*	**0.90149**	**0.69061**	0.56754	0.05186		0.00540
*P. a. aemodius*	**0.89086**	**0.68416**	**0.56089**	0.12339	−0.04375	

Note

Significant values shown in bold.

Significance level after Bonferroni correction *p* < .05/15 = .0034.

**FIGURE 6:**
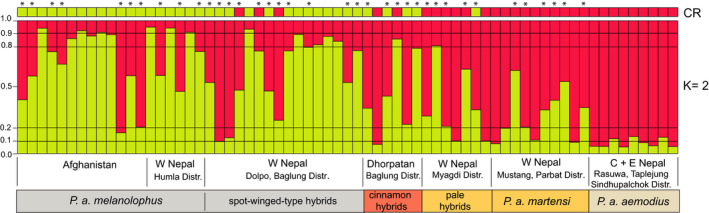
Genetic variation of Himalayan coal tit populations (*Periparus ater*; *n* = 67) based on 10 microsatellite loci. STRUCTURE analysis under the admixture‐frequency‐correlated model without locpriors a priori defined, STRUCTURE plot for most plausible *K* = 2; threshold *q* > 0.8 for assignment of individuals to genetic clusters according to Randi (2008); colored bars above the plot indicate individual assignment to the western (*P. a. melanolophus*) and eastern (*P. a. aemodius*) mitochondrial lineage, respectively; bars below the plot indicate phenotypically distinct populations along the east‐west gradient; asterisks at the top highlight genetic hybrid individuals

The results from STRUCTURE analysis are shown in Figure 6. Under both the admixture–frequency‐correlated model and the LOCPRIOR model, Evanno's ∆K separated two clusters (K = 2) as the most plausible population structure. Admixture between these two groups was generally high in most populations. For assignment probabilities of q < 0.8 eastern *P. a. aemodius* was the only unadmixed population, whereas all populations west of Bagmati Pradesh (Rasuwa District, westernmost range limit of *P. a. aemodius*) showed signs of admixture between the two genetic clusters (Figure 6; the results were similar for the separate run based on eight loci; not shown). In fact, only a minority of individuals in western Nepal could be clearly assigned to the eastern lineage (4 out of 10 *P. a. martensi*, 2 out of 10 pale‐bellied hybrids, 1 out of 6 cinnamon‐bellied hybrids) or to the western lineage (5 out of 16 spot‐winged‐type hybrids). Even phenotypical *P. a. melanolophus* from Afghanistan (who represented an unadmixed mtDNA gene pool of the western lineage) showed evidence of strong introgression of eastern *P. a. aemodius* alleles for more than half of the sampling (7 out of 13; Figure 6).

**FIGURE 7:**
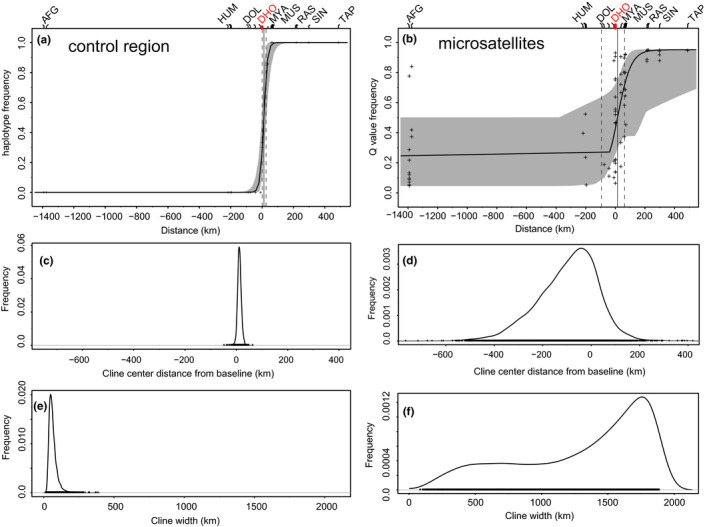
Cline analysis for Himalayan coal tits, *Periparus ater*. Based on mitochondrial CR sequences (a) shape, (c) center, (e) width and based on 10 microsatellite loci (b) shape, (d) center, (f) width; shape of the cline, (a, b): crosses indicate observed values (haplotype and allele frequencies) for each population; solid curves indicate maximum‐likelihood estimates of the cline with gray shapes indicating 95% CI of the estimates; solid lines indicate cline center estimates with dashed lines indication 95% CI of the estimates; major areas of origin indicated above: AFG, Afghanistan; HUM, Humla Distr.; DOL, Dolpo Distr.; DHO, Dhorpatan (Baglung Distr.); MYA, Myagdi Distr.; RAS, Rasuwa Distr.; SIN, Sindhu Palchok Distr.; TAP, Taplejung Distr

Both molecular data sets showed a clinal variation across the hybrid zone (Figure 7; Table 4), the null model had higher AICc values than those of all other cline models in the CR data set and than most cline models in the microsatellite data set (except three models). The best‐fit model for the mitochondrial CR data was model 1 from Derryberry et al. (2014) with *p*_min_ and *p*_max_ fixed to 0 and 1, respectively, at the tails of the cline (log likelihood = −0.740). For microsatellite data the best‐fit model was model 11 with observed *p*_min_ and *p*_max_ of 0.048 and 0.950, respectively (log likelihood = −8.220), reflecting a strong differentiation between westernmost *P. a. melanolophus* and easternmost *P. a. aemodius* anyway. Nevertheless, cline parameters differed significantly between the two data sets. The mean cline width estimate was 3.7 times larger for microsatellite data as compared to mitochondrial CR data (172 vs. 46 km). The center of the cline was estimated at 11 km east of Dhorpatan based on the CR data set and at 8 km east of Dhorpatan based on the microsatellite data set (for model and cline parameters see Table 4).

**TABLE 4:** Estimated cline shape parameters of best‐fit models for CR (haplotype frequencies; mtDNA) and 10 microsatellite loci (q score as inferred from STRUCTURE analysis for K = 2)

Data	*p*_min_	*p*_max_	Cline center (km)	Cline width (km)	logLike	Best‐fit model		
						No.	*p*_min_/*p*_max_	Tail fitting
CR	0.000	1.000	11 [0 – 25]	46 [24 – 95]	−0.740	1	Fixed	No fitting
Microsatellites	0.048	0.950	8 [−102 – 80]	172 [70 – 1040]	−8.220	11	Observed	Left tail only

Note

Estimates for frequencies at the western (*p*_min_) and eastern (*p*_max_) end of the cline, cline centers (95% CI of two log‐likelihood units in parenthesizes), cline widths (95% CI of two log‐likelihood units in parenthesizes) and log‐likelihood scores for fitted clines according to the best‐fit model selected based on the corrected AICc. Model number and scaling parameters according to Derryberry et al. (2014).

## 4 DISCUSSION

### 4.1 Location and characteristic of the Himalayan coal tit hybrid zone

Despite the outstanding species richness of the Himalayan biodiversity hotspot, phylogeographical patterns of the regional fauna are rather unexplored to date (review in Martens, 2015). So far, patterns of divergence and gene flow across zones of secondary overlap and contact in the Himalayas have been analyzed for a few conifer species (Poudel et al., 2012; Ryan et al., 2018) and for only two vertebrate species: wolves, *Canis lupus* (Werhahn et al., 2020) and greenish warblers, *Phylloscopus trochiloides* (Figure 2c; Alcaide et al., 2014).

The coal tit hybrid zone in western Nepal roughly corresponds to the Dhaulagiri transition zone of avian subspecies (Martens & Eck, 1995; Figure S1), where ranges of vicariant sister species may overlap, for example, those of Himalayan bush tits, *Aegithalos* (Figure 2b). While parental taxa and hybrids cooccur over wide areas of secondary overlap in other tit and chickadee taxa (e.g., great tits, *Parus major*, in the Middle Amur Valley: Kvist & Rytkönen, 2006; Fedorov et al., 2009; Figure 1a), the situation is different in Himalayan coal tits: The *P. ater* hybrid zone in Nepal is characterized by a chain of phenotypically diverse populations (Figure 1d) across an east‐west distance of roughly 100 km width (from the Dhorpatan Valley to easternmost records of *P. a. martensi* at Manang, Marsyandi Valley). The distinct color phenotypes of coal tit hybrids are confined to the same mountain valleys like, for example, distinct genetic lineages of Himalayan ground beetles in the catchments of Marsyandi Khola, Kali Gandaki and its side river valley Myagdi Khola (Schmidt et al., 2012). However, unlike in the ground beetle example, microallopatry does not seem to have triggered genetic diversification in the center of the Himalayan coal tit hybrid zone: Neither are populations genetically distinct, nor are the parameters of genetic variation lowered, as would be expected for small, isolated populations (Dixo et al., 2009; Frankham, 1996; Ortego et al., 2008). The main reason for this might be that mountain ridges provide less effective barriers to birds than to less mobile invertebrates, for example, flightless beetles (Schmidt et al., 2012) or scorpions of the North African Atlas Mountains (Habel et al., 2012). Accordingly, we found a signal of introgression of eastern *P. a. aemodius* microsatellite alleles even into westernmost populations of phenotypic *P. a. melanolophus*.

In several other avian hybrid zones, phenotypic clines were narrower than genetic clines (Hermansen et al., 2011; Kvist & Rytkönen, 2006; Poelstra et al., 2014), and allelic introgression might extend far beyond areas of, for example, vocal admixture (Sattler et al., 2007) or even across species boundaries (Kingston et al., 2014). With an estimated width of 46 km the mitochondrial cline across the Himalayan coal tit hybrid zone is rather narrow, because populations of *P. a. martensi* appeared to be unadmixed (only the eastern mtDNA lineage was present). That distance roughly corresponds to the east‐west extent of the European crow (*Corvus corone*) hybrid zone (Poelstra et al., 2013, 2014). In contrast, the microsatellite cline is about 3.7 times wider. This wide range of the hybrid zone is also reflected in the calculated divergence and diversity indices, that is, *F*_ST_ values confirmed a rather low diversification even between nonadjacent populations (Table 3). At the same time, the moderate heterozygote deficit indicates an ongoing admixture also in populations located at the margins of the hybrid zone. Taken together, and taking into account that wide clines indicate only weak selection against hybrids (Barton & Gale, 1993) and that microsatellites likely uncover more recent processes (Selkoe & Toonen, 2006), the Himalayan coal tit hybrid zone appears as a nonstable zone of ongoing hybridization and potentially continuing expansion.

### 4.2 Discordance of genetical clines

The microsatellite cline and the mitochondrial cline across the Himalayan coal tit range differ from each other. Not only is the microsatellite cline 3.7 times wider than the mitochondrial one (discordant clines), especially the left tail is clearly shallower in the microsattelite cline. Discongruity of clines inferred from different sets of molecular markes is a common phenomenon of terrestrial vertebrate hybrid zones: For several Nearctic avian taxon pairs, mitochondrial clines were about 1.9 to 20 times narrower than those inferred from neutral nuclear markers (Gowen et al., 2014; Kingston et al., 2012; Walsh et al., 2016). Similar discordance between mitochondrial and nuclear cline shapes was documented for Nearctic mule deer, *Odocoileus hemionus* (Haines et al., 2019) and Iberian Bosca's newts, *Lissotriton boscai* (Sequeira et al., 2020). The opposite pattern of microsatellite clines being narrower than mitochondrial clines seems to be less common (e.g., in the Japanese fire‐bellied newt, *Cynops pyrrhogaster*: Tominaga et al., 2018). More complex spatial patterns can even result in concordant and discordant clines for mtDNA and microsatellites across different hybrid zones of the same species, as shown for European grass snakes, *Natrix natrix* (Kindler et al., 2017) and the European pond turtle, *Emys orbicularis* (Pöschel et al., 2018; Vamberger et al., 2015). That great variation of terrestrial vertebrate hybrid zones in shape and extent is explained by a number of factors. For birds, comparisons among vertebrate classes suggested a positive correlation of hybrid zone width with dispersal abilities and mitochondrial DNA distance among parental taxa (McEntee et al., 2020). Sex‐biased dispersal in turn is one of several factors that might shape mitonuclear discordance of admixture patterns (Prugnolle & de Meeus, 2002) along with incomplete lineage sorting, differential drift (Bonnet et al., 2017; Toews & Brelsford, 2012), the particular mechanism of mutation of short tandem repeats such as microsatellites (Karl et al., 2012; Putman & Carbone, 2014) or purifying selection on mitochondrial markers (Morales et al., 2015). Another process that can cause discordant clines is hybrid zone movement (Taylor et al., 2014; van Riemsdijk et al., 2019; Wielstra et al., 2017). Since in hybrid zone movement one of the two hybridizing populations expands its distribution, the hybrid zone shifts while selectively neutral loci of the displaced population remain in the displacing population (Currat et al., 2008; Wielstra et al., 2017) and cause a tail of introgression in the wake of the hybrid zone (van Riemsdijk et al., 2019). This genetic footprint is thought to be primary reflected by microsatellite markers, as these are noncoding and have higher mutation rates than other markers (Ellegren, 2000, 2004). Indeed, introgression of microsatellite alleles seems to be asymmetric from eastern *P. a. aemodius* into western populations of phenotypic *P. a. melanolophus* but not vice versa. At the same time, significantly negative Tajima's D for western *P. a. melanolophus* and pale‐bellied hybrids might be another sign of an expanding population but may also be due to selection against mitochondrial markers (Tajima, 1989). Which of the named factors predominantly shape the clines and whether in fact movement or nondirectional expansion of the Himalayan coal tit hybrid zone is taking place remains to be further elucidated. After all, extent and directionality of introgression may depend on further factors like variation of morphological and behavioral traits such as passerine territorial song. Some of these might also help explaining the asymmetrical introgression of microsatellite alleles in Himalayan coal tits.

### 4.3 Asymmetric introgression across the hybrid zone

In vertebrates, asymmetric introgression between closely related taxa is often associated with differences in phenotype like in wall lizards (*Podacris muralis*; While et al., 2015; Yang et al., 2020), in particular when mate choice is related to differential body size such as in Nearctic woodrats, *Neotoma* sp. (Coyner et al., 2015) and the European pond turtle, *Emys orbicularis* (Pöschel et al., 2018; Vamberger et al., 2015). In birds, such a correlation between assortative mating, body size and differential introgression was found even in the rare case of female competition for mates in polyandrous tropical waders, *Jacana spinosa* and *J. jacana* (Lipshutz et al., 2019). Indeed, in the Himalayas the western and eastern parental taxa (*P. a. melanolophus* and *P. a. aemodius*) were shown to differ in body size and plumage proportions (Martens et al., 2006). Furthemore, in birds, sexually selective ornamental plumage traits can trigger directional mate choice, such as beneficial golden plumage in manakins, *Manacus* sp. (Parchman et al., 2013; Uy & Stein, 2007) and head coloration in white wagtails, *Motacilla alba* (Semenov et al., 2017) or *Ficedula* flycatchers (Haavie et al., 2000). In Australia, there is evidence of directional introgression of red‐plumage alleles across a hybrid zone of fairy wrens (*Malurus* sp., Baldassarre et al., 2014). Admixture patterns in a Nearctic warbler hybrid zone showed clustering of single‐nucleotide polymorphisms (SNPs) across parental genomes with candidate gene regions associated with color pigments, such as carotinoids or melanin (Brelsford et al., 2017). Likewise, in the Himalayan coal tit hybrid zone phenotypes of western and eastern parental taxa are highly distinctive (Figure 3), which might have facilitated assortative mating in past secondary contact prior to hybrid zone formation. This situation contrasts the European transition zone of the coal tit: There, phenotypes at both ends of a wide cline from the Iberian Peninsula towards Scandinavia show only subtle differences in plumage coloration (photographic images in Martens, 2012; for trans‐European patterns of genetic admixture see Tritsch et al., 2018). While there is evidence of an effect of ornamental plumage traits on assortative mating, for example, in the blue tit, *Cyanistes caeruleus* (Fargevieille et al., 2017; García‐Navas et al., 2009), this remains to be tested for the coal tit.

In songbirds (Oscines), territorial song plays a key role not only in territorial defense (intrasexual behavior) but also in mate choice (intersexual behavior; Naguib & Riebel, 2014; Päckert, 2018). Therefore, differences between song types facilitate assortative mating in secondary contact as shown for several oscine contact zones (*Ficedula* flycatchers in central Europe: Qvarnström et al., 2010; *Phylloscopus* leaf warblers in the Pyrenees: Helbig et al., 2001). Sexual selection acting on song types or larger and highly variable repertoires can lead to differences in mating success between parental taxa and thus lead to asymmetric gene flow across a hybrid zone. This was suggested for some hybridizing taxon pairs of tits and chickadees (great tit/ Japanese tit, *Parus major*/*P. minor*: Päckert et al., 2005; Kvist & Rytkönen, 2006; subspecies of the mountain chickadee, *Poecile gambeli*: Reudink et al., 2007; Manthey et al., 2012; Taylor et al., 2014). In playback experiments in a Nearctic titmice contact zone females of both species showed a clear preference for songs and phenotypes of the tufted titmouse, *Baeolophus bicolor*, and discriminated against those of the syntopic black‐crested titmouse, *B. atricristatus* (Curry & Patten, 2016). Similar asymmetries have been described for male aggression, such as in the hybrid area of hermit and Townsend's warbler (*Dendroica occidentalis*, *D. townsendi*; Pearson & Rohwer, 2000).

At a similar level of genetic divergence, the coal tit does not show strong diversification of song patterns among European populations and their Asian relatives (Pentzold et al., 2016; Tietze et al., 2011). Such uniformity of vocal patterns strongly contrasts strong divergence of European and Asian song structures in other tit species (groups), such as the great tits, *Parus major* (Päckert et al., 2005) or willow tits, *Poecile montanus* (Martens et al., 2003; Tritsch et al., 2017). Though Tietze et al. (2011) found subtle differences in maximum frequencies and element number among Himalayan song types of western *P. a. melanolophus* and eastern *P. a. aemodius*, variation of song types does not seem to affect species recognition in the coal tit. Playback experiments with Himalayan test birds suggested that the latter two subspecies mutually understand local song types and discriminated these strongly against European coal tit song and great tit song from Afghanistan (Martens, 1975: pp. 417–421). Therefore, vocalizations might be a less effective premating barrier for the Himalayan coal tits as compared with hybrid zones among other tit taxon pairs (Kvist & Rytkönen, 2006; Manthey et al., 2012; Taylor et al., 2014) or among leaf warbler species with strongly distinctive song types (Helbig et al., 2001; Shipilina et al., 2017; see Zhang et al., 2019 for a scenario of strong introgression among Eastern Himalayan leaf warbler taxa with less distinctive song types).

## 5 CONCLUSIONS

The existence of putative hybrid populations in the central Himalayas was the main argument for inclusion of all Himalayan taxa in one species‐level taxon, *Periparus ater*, under the Biospecies Concept (BSC). In contrast, the western Himalayan “spot‐winged tit” was often treated as a species of its own, *Periparus melanolophus* (Dickinson, 2003; Gosler & Clement, 2007; Vaurie, 1959) based on its distinctiveness in plumage coloration and thus according to the diagnosability criterion of the Phylogenetic Species Concept (PSC) (Sangster, 2014). This mere typological approach was already challenged by Päckert and Martens (2008) who outlined two major problems that resulted from earlier molecular studies (Martens et al., 2006): (i) paraphyly of a least‐inclusive species‐level taxon *P. ater* excluding the form *melanolophus* (in conflict with the PSC; confirmed by Päckert et al., 2011; Tietze et al., 2011), (ii) the existence of putative hybrid populations in the central Himalayas (in conflict with the BSC). However, the putative hybrid origin of the central Himalayan populations from Myagdi District and Baglung District has not been verified by any comprehensive population genetic analysis to date. Our results shed new light on the Himalayan coal tits hybrid zone showing strong genetic admixture of the putative phenotypic hybrid populations (both marker systems) that overcome the phenotypic pattern of microallopatry. While we thus could confirm our first hypothesis of strong genetic admixture of phenotypically intermediate populations, the second hypothesis of genetic distinctiveness of potential parental populations has to be rejected (at least on the basis of a limited number of microsatellite loci) as introgression of eastern alleles even extends beyond the range of phenotypic hybrids into the western parental form *P. a. melanolophus*. This is in good accordance with the current consent among taxonomist on the inclusion of this western Himalayan taxon in one species‐level taxon *Periparus ater* (Gill et al., 2020; del Hoyo et al., 2016).

Despite all reservations against inference of admixture proportions from microsatellite data (Balloux et al., 2000; Lemopoulos et al., 2019; Putman & Carbone, 2014), microsatellite data sets performed equally well for detection of patterns of divergence and admixture as genome‐wide SNPs in several studies (Fernández et al., 2013; Ljungqvist et al., 2010; Narum et al., 2008; Roques et al., 2019). However, additional markers offer a chance for a better small‐scale resolution of the phylogeographical structure in the center of the Himalayan hybrid zone. By this, they are a perspective to better understand the putative diversification patterns, for example, among the distinctive cinnamon‐bellied hybrids and other phenotypes. Whole‐genome data might also provide a deeper insight into processes causing asymmetric introgression and shaping the Himalayan coal tit hybrid zone as one of the rare examples of a genetically well‐defined avian hybrid zone in the Himalayas.

## CONFLICT OF INTEREST

The authors declare no conflicts of interest.

## AUTHOR CONTRIBUTIONS

**Hannes Wolfgramm:** Formal analysis (lead); Investigation (equal); Methodology (equal); Validation (equal); Visualization (equal); Writing‐original draft (equal); Writing‐review & editing (equal). **Jochen Martens:** Conceptualization (equal); Data curation (equal); Funding acquisition (equal); Investigation (equal); Resources (equal); Writing‐review & editing (equal). **Till Töpfer:** Data curation (equal); Resources (equal); Writing‐review & editing (equal). **Melita Vamberger:** Formal analysis (equal); Investigation (equal); Methodology (equal); Software (equal); Supervision (equal); Writing‐review & editing (equal). **Abhinaya Pathak:** Resources (equal); Supervision (equal); Validation (equal); Writing‐review & editing (equal). **Heiko Stuckas:** Conceptualization (equal); Investigation (equal); Methodology (equal); Software (equal); Supervision (equal); Validation (equal); Writing‐review & editing (equal). **Martin Päckert:** Conceptualization (equal); Data curation (lead); Investigation (lead); Project administration (lead); Resources (equal); Supervision (equal); Validation (equal); Visualization (equal); Writing‐original draft (lead); Writing‐review & editing (equal).

## Supporting information

Supplementary Material

## Data Availability

CR sequences used in this study (inferred from previous studies and newly generated) are available at GenBank, accession numbers are listed in Appendix Tables S1 and S2. A data package including microsatellite allele lengths, the CR alignment and specimen and sample metadata is available for download at Dryad Digital Repository (https://doi.org/10.5061/dryad.0gb5mkm28).
