# Supplementary material for: Asymmetric allelic introgression across a hybrid zone of the coal tit (Periparus ater) in the central Himalayas
Source: Ecol Evol. 2021 Nov 24;11(23):17332–51. doi: 10.1002/ece3.8369 (PMC8668783; doi:10.1002/ece3.8369)
Supplement: Supplementary file 1 — Supplementary Material [file ECE3-11-17332-s001.docx]

**Appendix – Supplementary Figures and Tables**


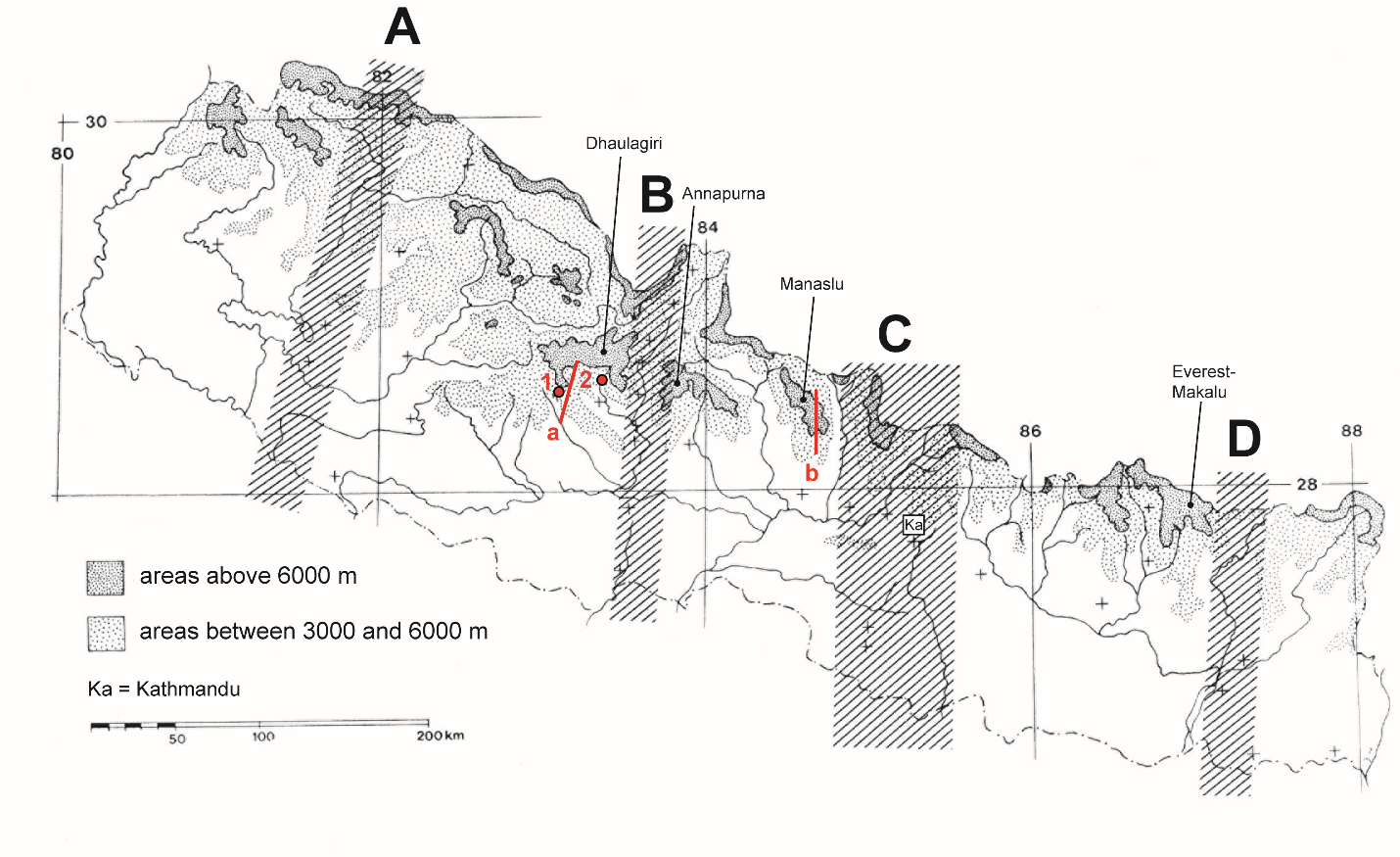


Appendix Figure S1: Subspecies transition areas in Nepal according to Martens & Eck (1995; modified from their Figs 41 and 42); A= Karnali catchment transition; B= Dhaulagiri transition; C= Kathmandu transition; D= Arun catchment transition; red lines indicate the approximate extent of the coal tit hybrid zone according to Eck & Martens (2006; Fig. 3) with a= eastern range limit of *P. m. melanolophus* and b= eastern range limit of *P. a. martensi*; red dots indicate coal tit hybrid populations: 1= cinnamon-bellied hybrids at Dhorpatan, 2= pale-bellied hybrids at Myagdi Khola.

Appendix Table S1: Information on individuals and populations through the transect from Peiwar, Afghanistan to Ghunsa, Taplejung District, Nepal. Distances were calculated as great circle geographical distances from Dhorpatan, Baglung District, Nepal; distances to localities west of Dhorpatan were defined as negative values; q scores inferred from STRUCTURE analysis with 10 microsatellite loci for K=2 and CR haplotype inferred from haplotype network; CR freq are mean CR haplotype frequencies per population (0 = only western haplotypes, 1 = only eastern haplotypes); GB acc no: Genbank accession numbers; specimen ID = catalogue number of voucher specimens; collection acronyms: ZFMK = Zoologisches Forschungsmuseum Alexander Koenig, Bonn; NME = Naturkundemuseum Erfurt (voucher specimen, no ID available for some specimens); MTDC = Senckenberg Naturhistorische Sammlungen, Museum für Tierkunde Dresden.

| **sample ID** | **Specimen ID** | **GB acc no** | **taxon** | **country** | **locality** | **distance**  **(km)** | **q score** | **CR haplotype** | **CR freq** |
| --- | --- | --- | --- | --- | --- | --- | --- | --- | --- |
| MAR8322 | ZFMK Niethammer536 | OK358028 | *P. a. melanolophus* | Afghanistan | Peiwar | -1391.36 | 0.056 | mela1 | 0 |
| MAR8323 | ZFMK Niethammer582 | OK358029 | *P. a. melanolophus* | Afghanistan | Peiwar | -1391.36 | 0.233 | mela1 |  |
| MAR8324 | ZFMK Niethammer610 | OK358030 | *P. a. melanolophus* | Afghanistan | Peiwar | -1391.36 | 0.327 | mela1 |  |
| MAR8325 | ZFMK Niethammer545 | OK358031 | *P. a. melanolophus* | Afghanistan | Peiwar | -1391.36 | 0.135 | mela2 |  |
| MAR8326 | ZFMK Niethammer595 | OK358032 | *P. a. melanolophus* | Afghanistan | Peiwar | -1391.36 | 0.075 | mela1 |  |
| MAR8327 | ZFMK Niethammer571 | OK358033 | *P. a. melanolophus* | Afghanistan | Peiwar | -1391.36 | 0.115 | mela6 |  |
| MAR8328 | ZFMK Niethammer580 | OK358034 | *P. a. melanolophus* | Afghanistan | Peiwar | -1391.36 | 0.087 | mela1 |  |
| MAR8329 | ZFMK Niethammer537 | OK358035 | *P. a. melanolophus* | Afghanistan | Peiwar | -1391.36 | 0.105 | mela1 |  |
| MAR8332 | ZFMK Niethammer578 | OK358038 | *P. a. melanolophus* | Afghanistan | Peiwar | -1391.36 | 0.8 | mela1 |  |
| MAR8333 | ZFMK Niethammer581 | OK358039 | *P. a. melanolophus* | Afghanistan | Peiwar | -1391.36 | 0.048 | mela1 |  |
| MAR8330 | ZFMK Niethammer258 | OK358036 | *P. a. melanolophus* | Afghanistan | Nuristan | -1375.57 | 0.843 | mela1 | 0 |
| MAR8331 | ZFMK Niethammer247 | OK358037 | *P. a. melanolophus* | Afghanistan | Nuristan | -1375.57 | 0.414 | mela7 |  |
| MTDC52891 | MTDC52891 | OK358005 | *P. a. melanolophus* | Afghanistan | Nuristan | -1375.57 | 0.416 | mela1 |  |
| MAR2913 | NME | JX474770 | *P. a. melanolophus* | Nepal | Chala | -217.77 | 0.412 | mela1 | 0 |
| MAR2921 | NME | JX474771 | *P. a. melanolophus* | Nepal | Simikot | -202.39 | 0.532 | mela1 | 0 |
| MAR3324 |  | DQ466209 | *P. a. melanolophus* | Nepal | Chucho Kohla | -201.82 | 0.232 | mela9 |  |
| MAR2918 | NME 01/024 | JF304545 | *P. a. melanolophus* | Nepal | Saipal | -195.7 | 0.055 | mela1 | 0 |
| MAR6615 | NME | JX474779 | *P. a. melanolophus* | Nepal | Jagdula. Lekh | -88.8 | --- | mela8 | 0 |
| MAR8316 | ZFMK 95.026 | OK358022 | spot-winged-type hybrids | Nepal | Ringmo | -75.41 | 0.2 | mela1 | 0 |
| MAR8317 | ZFMK 74.416 | OK358023 | spot-winged-type hybrids | Nepal | Gompa | -42.8 | 0.18 | mela3 | 0 |
| MAR8318 | ZFMK 74.417 | OK358024 | spot-winged-type hybrids | Nepal | Gompa | -42.8 | 0.118 | mela1 |  |
| MAR8308 | ZFMK 74.425 | OK358014 | spot-winged-type hybrids | Nepal | Thankur | -9.37 | 0.881 | mela5 | 0 |
| MAR8314 | ZFMK 74.414 | OK358020 | spot-winged-type hybrids | Nepal | Thankur | -9.37 | 0.227 | mela1 |  |
| MAR8315 | ZFMK 74.415 | OK358021 | spot-winged-type hybrids | Nepal | Thankur | -9.37 | 0.103 | mela1 |  |
| MAR8306 | ZFMK 2006/53 | OK358012 | spot-winged-type hybrids | Nepal | Dhorpatan | 0 | 0.465 | mela1 | 0.3 |
| MAR8307 | ZFMK 2006/55 | OK358013 | spot-winged-type hybrids | Nepal | Dhorpatan | 0 | 0.909 | mela1 |  |
| MAR8309 | ZFMK 74.408 | OK358015 | spot-winged-type hybrids | Nepal | Dhorpatan | 0 | 0.522 | aem1 |  |
| MAR8310 | ZFMK 74.409 | OK358016 | spot-winged-type hybrids | Nepal | Dhorpatan | 0 | 0.064 | mela4 |  |
| MAR8311 | ZFMK 74.410 | OK358017 | spot-winged-type hybrids | Nepal | Dhorpatan | 0 | 0.228 | aem3 |  |
| MAR8312 | ZFMK 74.411 | OK358018 | spot-winged-type hybrids | Nepal | Dhorpatan | 0 | 0.529 | mela1 |  |
| MAR8313 | ZFMK 74.412 | OK358019 | spot-winged-type hybrids | Nepal | Dhorpatan | 0 | 0.749 | aem2 |  |
| MAR8319 | ZFMK 74.418 | OK358025 | spot-winged-type hybrids | Nepal | Dhorpatan | 0 | 0.155 | mela1 |  |
| MAR8320 | ZFMK 74.419 | OK358026 | spot-winged-type hybrids | Nepal | Dhorpatan | 0 | 0.465 | mela1 |  |
| MAR8321 | ZFMK 74.420 | OK358027 | spot-winged-type hybrids | Nepal | Dhorpatan | 0 | 0.225 | mela1 |  |
| MAR8301 | ZFMK 74.443 | OK358007 | cinnamon-bellied hybrids | Nepal | Dhorpatan | 0 | 0.931 | aem1 | 0.34 |
| MAR8302 | ZFMK 95.025 | OK358008 | cinnamon-bellied hybrids | Nepal | Dhorpatan | 0 | 0.567 | mela1 |  |
| MAR8303 | ZFMK 2006/42 | OK358009 | cinnamon-bellied hybrids | Nepal | Dhorpatan | 0 | 0.138 | aem4 |  |
| MAR8304 | ZFMK 2006/54 | OK358010 | cinnamon-bellied hybrids | Nepal | Dhorpatan | 0 | 0.78 | mela1 |  |
| MAR8305 | ZFMK 74.442 | OK358011 | cinnamon-bellied hybrids | Nepal | Dhorpatan | 0 | 0.207 | mela1 |  |
| MTDC58572 | MTDC58572 | OK358006 | cinnamon-bellied hybrids | Nepal | Dhorpatan | 0 | 0.658 | mela1 |  |
| MAR90155 | ZFMK_ORN 2006.45 | JX474786 | pale-bellied hybrids | Nepal | Myagdi Khola | 37.26 | 0.188 | aem1 | 0.86 |
| MAR90156 | ZFMK_ORN 2006.46 | JX474787 | pale-bellied hybrids | Nepal | Myagdi Khola | 37.26 | 0.795 | aem1 |  |
| MAR90157 | ZFMK_ORN 2006.47 | JX474788 | pale-bellied hybrids | Nepal | Myagdi Khola | 37.26 | 0.905 | aem1 |  |
| MAR90163 | ZFMK_ORN 2006.49 | JX474789 | pale-bellied hybrids | Nepal | Myagdi Khola | 37.26 | 0.365 | aem1 |  |
| MAR90164 | ZFMK_ORN 2006.50 | JX474790 | pale-bellied hybrids | Nepal | Myagdi Khola | 37.26 | 0.717 | aem1 |  |
| MAR90165 | ZFMK_ORN 2006.48 | DQ466219 | pale-bellied hybrids | Nepal | Myagdi Khola | 37.26 | 0.67 | mela1 |  |
| MAR90166 | ZFMK_ORN 2006.51 | JX474791 | pale-bellied hybrids | Nepal | Myagdi Khola | 37.26 | 0.905 | aem1 |  |
| MAR8335 | ZFMK 74,470 | OK358041 | *P. a. martensi* | Nepal | Nabrikot Khola | 56.93 | 0.809 | aem1 | 1 |
| MAR8336 | ZFMK 74.459 | OK358042 | *P. a. martensi* | Nepal | Kali Gandaki Valley | 60.83 | 0.374 | aem1 |  |
| MAR8338 | ZFMK 74.467 | OK358044 | *P. a. martensi* | Nepal | Kali Gandaki Valley | 60.83 | 0.898 | aem1 |  |
| MAR8340 | ZFMK 2006/41 | OK358046 | *P. a. martensi* | Nepal | Kali Gandaki Valley | 60.83 | 0.598 | aem1 |  |
| MAR90132 | - | DQ466218 | *P. a. martensi* | Nepal | Purano Marpha | 66.59 | 0.915 | aem2 | 1 |
| MAR90137 | ZFMK_ORN 2006.44 | JX474785 | *P. a. martensi* | Nepal | Purano Marpha | 66.59 | 0.654 | aem1 |  |
| MAR8334 | ZFMK 74.468 | OK358040 | *P. a. martensi* | Nepal | Thakkhola | 67.48 | 0.925 | aem2 |  |
| MAR8337 | ZFMK 74.469 | OK358043 | *P. a. martensi* | Nepal | Thakkhola | 67.48 | 0.8 | aem1 |  |
| MAR8339 | ZFMK 74.465 | OK358045 | *P. a. martensi* | Nepal | Thakkhola | 67.48 | 0.671 | aem2 |  |
| MAR90101 | ZFMK_ORN 2006.43 | DQ466217 | *P. a. martensi* | Nepal | Marsyandi | 70.94 | 0.457 | aem3 |  |
| MAR4155 | - | JX474773 | *P. a. aemodius* | Nepal | Somdang | 213.4 | 0.946 | aem1 | 1 |
| MAR4156 | - | DQ466214 | *P. a. aemodius* | Nepal | Somdang | 213.4 | 0.888 | aem1 |  |
| MAR4222 | - | JX474777 | *P. a. aemodius* | Nepal | Somdang,  4. & 8. camp | 213.4 | 0.872 | aem2 |  |
| MAR4196 | - | JX474776 | *P. a. aemodius* | Nepal | Somdang,  6. camp | 221.32 | 0.95 | aem2 |  |
| MAR4195 | - | JX474774 | *P. a. aemodius* | Nepal | Somdang | 221.32 | --- | aem1 |  |
| MAR90018 | - | JX474782 | *P. a. aemodius* | Nepal | Dadar Danda | 298.03 | --- | aem5 | 1 |
| MAR90019 | - | DQ466215 | *P. a. aemodius* | Nepal | Dadar Danda | 298.03 | 0.918 | aem6 |  |
| MAR90028 | - | JX474783 | *P. a. aemodius* | Nepal | Dadar Danda | 298.03 | 0.941 | aem8 |  |
| MAR90046 | - | JX474784 | *P. a. aemodius* | Nepal | Dadar Danda | 298.03 | 0.877 | aem2 |  |
| MAR90053 | - | DQ466216 | *P. a. aemodius* | Nepal | Dadar Danda | 298.03 | 0.947 | aem7 |  |
| MAR3681 | NME | JX474772 | *P. a. aemodius* | Nepal | Ghunsa | 487.02 | 0.945 | aem1 | 1 |

Appendix Table S2: CR sequences representing the Chinese and the Northeasterrn/Central Palearctic mitochondrial lineages of the coal tit (*Periparus ater*) used for reconstruction of haplotype networks.

| **sample ID** | **GB acc no** | **taxon** | **country** | **locality** | **CR haplotype** |
| --- | --- | --- | --- | --- | --- |
| MAR1416 | DQ466210 | *P. a. ater* | Russia | Sakhalin, Chaplanovo | ater5 |
| MAR1444 | JX170069 | *P. a. ater* | Russia | Primorye, Vladivostok | ater1 |
| MAR1445 | JX170070 | *P. a. ater* | Russia | Primorye, Vladivostok | ater2 |
| MAR1446 | DQ466205 | *P. a. ater* | Russia | Primorye, Vladivostok | ater6 |
| MAR1447 | JX170072 | *P. a. ater* | Russia | Primorye, Vladivostok | ater4 |
| MAR1448 | JX170073 | *P. a. ater* | Russia | Primorye, Vladivostok | ater1 |
| MAR1449 | JX170074 | *P. a. ater* | Russia | Primorye, Vladivostok | ater1 |
| MAR1450 | JX170075 | *P. a. ater* | Russia | Primorye, Vladivostok | ater3 |
| MAR1451 | JX170076 | *P. a. ater* | Russia | Primorye, Vladivostok | ater2 |
| MAR1452 | JX170077 | *P. a. ater* | Russia | Primorye, Vladivostok | ater3 |
| MAR1471 | DQ466211 | *P. a. ater* | Russia | Londoko | ater1 |
| MAR90028 | JX170079 | *P. a. insularis* | Japan | Hokkaido | ater1 |
| MAR90029 | JX170080 | *P. a. insularis* | Japan | Hokkaido | ater2 |
| MAR1608 | JX170063 | *P. a. rufipectus* | Kazakhstan | Almaatinka Valley | ater1 |
| MAR2878 | DQ466206 | *P. a. rufipectus* | Kyrgyzstan | Issyk-Kul | ater7 |
| MAR2879 | JX474770 | *P. a. rufipectus* | Kyrgyzstan | Issyk-Kul | ater1 |
| MAR2880 | JX474771 | *P. a. rufipectus* | Kyrgyzstan | Issyk-Kul | ater1 |
| MAR2881 | DQ466209 | *P. a. rufipectus* | Kyrgyzstan | Issyk-Kul | ater1 |
| MAR1876 | JF304545 | *P. a. eckodedicatus* | China | Gansu | ecko2 |
| MAR1880 | JX474779 | *P. a. eckodedicatus* | China | Gansu | ecko1 |
| MAR3242 | DQ466212 | *P. a. eckodedicatus* | China | Yunnan | ecko3 |
| MAR3280 | JX474775 | *P. a. eckodedicatus* | China | Sichuan | ecko1 |
| MAR4934 | JX474778 | *P. a. eckodedicatus* | China | Shaanxi | ecko5 |
| MAR5724 | JX474780 | *P. a. kuatunensis* | China | Jiangxi | ecko6 |
| MAR6902 | JX474781 | *P. a. eckodedicatus* | China | Ningxia | ecko1 |
| MAR6903 | DQ466212 | *P. a. eckodedicatus* | China | Ningxia | ecko1 |
| MAR8535 | OK358049 | *P. a. eckodedicatus* | China | Yunnan | ecko4 |

Appendix Table S3: Characteristics and variation of thirteen microsatellite loci used for population genetic analysis of coal tits. Selection of loci according to (Tritsch *et al.*, 2018); fluor. label = fluorescent label of forward primer, multiplex = allocation to multiplex set, Tm = primer pair annealing temperature.

| Locus | Primer sequence 5’-3’ | Fluor. label | Multiplex | Repeat motif | T_m_ (°C) | Allele size (bp) |
| --- | --- | --- | --- | --- | --- | --- |
| Parate01 | F: TCCTGGAGCACATTATGTCTATG | HEX | 2 | (TAGA)_14_  (CAGA)_4_ | 56 | 209 - 271 |
|  | R: AATCTGCTGCTCCATACTTGG |  |  |  |  |  |
| Parate02 | F: AGGGACAGAATTGTGCAAGG | HEX | 1 | (ATCT)_14_ | 56 | 189 - 373 |
|  | R: TGCATTCATGCATACATAGACAC |  |  |  |  |  |
| Parate03 | F: TGTTGTCTGCAAAAGGCAAG | 6-FAM | 1 | (ATAG)_14_ | 56 | 117 - 177 |
|  | R: CAAAGCCTTCATCTGCTTGG |  |  |  |  |  |
| Parate06 | F: TTCAGTGCAGGTGCATAATTG | 6-FAM | 1 | (CTAT)_15_ | 56 | 219 - 355 |
|  | R: GGCCAAGAGAAGTAGGGTGTAG |  |  |  |  |  |
| Parate07 | F: CTCCCAAGAGAGTCTGTGTCG | Atto 550 | 1 | (CTAT)_12_ | 56 | 166 - 199 |
|  | R: AAGGCTTTTGAAACAGGAGAAG |  |  |  |  |  |
| Parate08 | F: TTGTAACGACCTTGCACCTC | HEX | 1 | (CA)_20_ | 50 | 90 - 153 |
|  | R: AGGCAGTAAAACCCTCATGG |  |  |  |  |  |
| Parate09 | F: GGCACAGATGCATATTTTGTTTAC | HEX | 2 | (GT)_13_ | 56 | 122 - 136 |
|  | R: TGCACAATCATGCTTAATCCTC |  |  |  |  |  |
| Parate15 | F: TCACAAAAAGGCATTTGCAG | 6-FAM | 2 | (TC)_12_(C)_4_(TC)_7_CC(TC)_4_ | 56 | 129 - 204 |
|  | R: GGAGACAGGAGAGCAGCAAC |  |  |  |  |  |
| Parate16 | F. CTTTCTTGAATGCTCAGATTGC | Atto 550 | 2 | (CT)_27_ | 56 | 166 - 263 |
|  | R: CAAGCCCATGTTCAAGGTTC |  |  |  |  |  |
| Pat2-43 | F: ACAGGTAGTCAGAAATGGAAAG | Atto 565 | 1 | (CT)n | 60 | 126 - 213 |
|  | R: GTATCCAGAGTCTTTGCTGATG |  |  |  |  |  |
| PmaTGAn33 | F: TTCCCCAAGTATCCTGCATC | Atto 565 | 1 | (GATA)14GAT(GATA)8 | 57 | 258 - 398 |
|  | R: AAACCATATCACCCAGTGCC |  |  |  |  |  |
| Pma69 | F: CCCAGACAAAGCATCACTGG | Atto 565 | 2 | (TG)6 | 57 | 214 – 222 |
|  | R: GACAGTTCACATAGCCCTGG |  |  |  |  |  |
| PmaC25 | F: CGTCCTGCTGTTTGTATTTCTG | 6-FAM | 2 | (GAT)11 | 57 | 313 – 349 |
|  | R: CCATGAACCATTTTTAGGGTG |  |  |  |  |  |

Appendix Table S4: Characteristics of microsatellite loci per locus and population: Observed and expected heterozygosity (H_O_, H_E_), departure from Hardy-Weinberg equilibrium (p-value), potential indications of experimental failures (na = null alleles, sb = stutter bands) and pairwise linkage disequilibrium of loci (LD). Bonferroni corrected p-value for HWE p < 0.05/10 = 0.005 (departure from equilibrium is indicated in bold); Bonferroni corrected p-value for LD p < 0.05/45 = 0.00076.

| **Locus** | **Parate01** | **Parate02** | **Parate03** | **Parate06** | **Parate07** | **Parate08** | **Parate09** | **Parate15** | **Parate16** | **Pat2-43** |
| --- | --- | --- | --- | --- | --- | --- | --- | --- | --- | --- |
| ***P. a. melanolophus* West (n= 19)** | | | | | | | | | | |
| H_O_ | 1.000 | 0.737 | 0.789 | 0.474 | 0.684 | 0.526 | 0.737 | 0.474 | 0.211 | 0.053 |
| H_E_ | 0.853 | 0.868 | 0.832 | 0.838 | 0.771 | 0.930 | 0.718 | 0.642 | 0.650 | 0.053 |
| p-value | 0.755 | 0.157 | 0.783 | **0.0003** | **< 0.001** | **< 0.001** | 0.438 | 0.192 | **< 0.001** | 1.000 |
| exp. failure |  |  |  | sb, na |  | na |  |  | sb, na |  |
| LD | Parate02 – Parate03; Parate03 – Parate09 | | | | | | | | | |
| **spot-winged-type hybrids (n= 16)** | | | | | | | | | | |
| H_O_ | 0.688 | 0.643 | 0.938 | 0.500 | 0.875 | 0.500 | 0.438 | 0.688 | 0.250 | 0.625 |
| H_E_ | 0.823 | 0.841 | 0.780 | 0.897 | 0.837 | 0.879 | 0.766 | 0.702 | 0.675 | 0.554 |
| p-value | 0.199 | 0.126 | 0.490 | **< 0.001** | 0.229 | **0.002** | 0.005 | 0.559 | **0.0004** | 0.805 |
| exp. failure |  | na |  | na |  | na | na |  | na |  |
| LD | Parate02 – Parate03; Parate02 – Parate06; Parate02 – Parate15 | | | | | | | | | |
| **cinnamon-bellied hybrids (n=6)** | | | | | | | | | | |
| H_O_ | 1.000 | 0.667 | 0.833 | 0.500 | 1.000 | 0.167 | 0.667 | 1.000 | 0.500 | 0.500 |
| H_E_ | 0.955 | 0.909 | 0.803 | 0.848 | 0.879 | 0.864 | 0.727 | 0.788 | 0.682 | 0.455 |
| p-value | 1.000 | 0.113 | 0.345 | 0.164 | 0.825 | **< 0.001** | 0.513 | 1.000 | 0.544 | 1.000 |
| exp. failure |  |  |  |  |  | na |  |  |  |  |
| LD | --- | | | | | | | | | |
| **pale-bellied hybrids (n=7)** | | | | | | | | | | |
| H_O_ | 1.000 | 1.000 | 1.000 | 1.000 | 1.000 | 0.857 | 0.857 | 0.714 | 0.714 | 0.571 |
| H_E_ | 0.945 | 0.939 | 0.791 | 0.901 | 0.824 | 0.846 | 0.747 | 0.670 | 0.659 | 0.681 |
| p-value | 1.000 | 1.000 | 1.000 | 1.000 | 1.000 | 0.518 | 0.89 | 1.000 | 1.000 | 0.220 |
| exp. failure |  |  |  |  |  |  |  |  |  |  |
| LD | --- | | | | | | | | | |
| ***P. a. martensi* (n=10)** | | | | | | | | | | |
| H_O_ | 0.900 | 0.778 | 0.700 | 0.800 | 0.900 | 0.600 | 0.400 | 0.700 | 0.500 | 0.600 |
| H_E_ | 0.947 | 0.948 | 0.821 | 0.868 | 0.763 | 0.905 | 0.763 | 0.742 | 0.695 | 0.637 |
| p-value | 0.551 | 0.142 | 0.249 | 0.197 | 0.859 | **< 0.001** | 0.006 | 0.103 | 0.375 | 0.501 |
| exp. failure |  |  |  |  |  |  |  |  |  |  |
| LD | --- | | | | | | | | | |
| ***P. a. aemodius* (n=9)** | | | | | | | | | | |
| H_O_ | 0.889 | 1.000 | 0.667 | 0.444 | 0.889 | 0.333 | 0.889 | 1.000 | 0.889 | 0.778 |
| H_E_ | 0.922 | 0.922 | 0.830 | 0.922 | 0.784 | 0.895 | 0.837 | 0.784 | 0.830 | 0.837 |
| p-value | 0.220 | 1.000 | 0.321 | **< 0.001** | 0.339 | **0.0003** | 0.936 | 0.444 | 0.518 | 0.189 |
| exp. failure |  |  |  | na |  | na |  |  |  |  |
| LD | Parate02 – Parate03; Parate03 – Parate08; Parate15 – Pat2-43 | | | | | | | | | |
